# Obesity: An Instagram Analysis

**DOI:** 10.7759/cureus.39619

**Published:** 2023-05-28

**Authors:** Umme Aiman, Maneeth Mylavarapu, Namra V Gohil, Shubham Holge, Ashwin Gajre, Kodali Akhila, Nitin M Ghadge

**Affiliations:** 1 Department of Internal Medicine, Bhaskar Medical College and Hospitals, Hyderabad, IND; 2 Department of Public Health, Adelphi University, Garden City, USA; 3 Internal Medicine, Medical College Baroda, Vadodara, IND; 4 Internal Medicine, Government Medical College and Hospital, Sindhudurg, IND; 5 Internal Medicine, Lokmanya Tilak Municipal Medical College, Mumbai, IND; 6 Medicine, Andhra Medical College, Visakhapatnam, IND; 7 Communicable Disease, New York State Department of Health, Albany, USA

**Keywords:** healthcare information., digital health, social media, instagram, obesity

## Abstract

Introduction

Although the pathophysiology of obesity is widely recognized, its psychological and social aspects have received more attention in treatment and prevention. Social media technological advancements offer a quicker, more accessible, and broader platform for the dissemination of information. Hence, social media may significantly impact eating habits and body image development in children and adolescents, potentially turning into a risk factor for obesity if the behaviors being pushed are not consistent with a healthy lifestyle.

Aim

This study aims to evaluate the quality and reliability of content being circulated on Instagram related to the disease "obesity."

Methods

A cross-sectional observational study was conducted virtually over a period of ten days. Six hashtags related to the disease "obesity" were screened. Posts in the languages "English" or "Hindi" containing information about the disease "obesity" were included in the study. A questionnaire was made to assess these posts based on various pre-determined categories: type of post, type of information circulated, quality, reliability, and correctness.

Results

After applying the inclusion criteria, 420 posts were included in our study. 84% of the relevant posts were images/posts, and 15% were videos. Only 17% were posted by doctors, while the health and wellness industry posted around 54.52%. Survivors/persons suffering from the disease contributed to 13.81%, while that from dietitians was 6.43%, and that from new agencies was only 1.19%. The number of correct posts by doctors, nurses, and hospitals was 54.93%, and others were 37.7%. The posts by doctors, nurses, and hospitals were more reliable compared to others (statistically significant p<0.05).

Conclusion

This study highlights the need for continued monitoring and evaluation of the social media platform Instagram for the spread of healthcare information.

## Introduction

Obesity is identified using the body mass index (BMI), which is calculated by dividing weight in kilograms by height in square meters. As per Asian-Pacific guidelines, adults with a BMI between 23.0 and 24.9 kg/m^2^ are considered overweight, and those with a BMI of 25 kg/m^2^ or greater are considered obese [1]. There were 2.1 billion people who were classified as overweight or obese globally between 1980 and 2013, a rise of 27.5% for adults and a 47.1% increase for children.

Obesity is multifactorial, involving complex relationships among biological, psychological, and behavioral factors such as genetic makeup, socioeconomic situation, and cultural influences. Although the pathophysiology of obesity is widely recognized, the disease's psychological and social aspects have received more attention in treatment and prevention [2].

Social media technological developments offer a quicker, more accessible, and wider means of dissemination. Based on emotional reactions to various information inputs, the emotional interactions that users have while utilizing these platforms will have a dynamic impact on their thoughts and actions [3]. Social media are certainly in the service of health, following it with their information, advice, statistics, discoveries, and innovations [4]. Social media can have a significant impact on how children and adolescents develop their eating habits and body image, which may be one of the risk factors for obesity if the behaviors being pushed are not consistent with a healthy lifestyle. It is also important to note that social media can be a useful tool for both preventing and treating obesity [5]. This study aims to assess the type of information circulated about "obesity" by categorizing it into symptoms and treatment, and to evaluate this information for quality, reliability, and authenticity.

## Materials and methods

An observational study of the cross-sectional type was conducted over a period of ten days, virtually, in December 2022. To maintain homogenous data, Instagram, a social media platform largely used by the young adult population, was used to assess the information available about "obesity." The top seven hashtags in "obesity," namely #Obesity, #Stopobesity, #Obesityfreeindia, #Obesitysurgery, #Obesityawareness, #Obesitysurvivor, and #Fightingobesity, were taken into this study. Posts in the languages "English" or "Hindi" containing information about "Obesity" were included in the study. All other posts were excluded. Each author was allotted one hashtag, and they analyzed ten "recent" posts every day for ten days.

A questionnaire with pre-determined categories was made for assessment of these posts based on: the type of post and the type of user who uploaded this post (doctor, health and wellness industry, and others); the type of information about "obesity" (descriptive, epidemiology, symptoms, preventive measures, treatment, etc.); and assessing if this information is correct or not. Posts that were factually correct as determined by the World Health Organization Factsheet on Hepatitis and CDC guidelines were deemed "true"; otherwise, they were "false." A total of 700 posts were analyzed, and 420 posts that met the inclusion criteria were included in this study. The quality of the posts was evaluated using the Global Quality Score (GQS), and reliability was evaluated using the Reliability Score (Tables 1-2). The data were collected in Microsoft Excel (Microsoft® Corp., Redmond, WA) and analyzed using SPSS software (IBM Corp., Armonk, NY).

**Table 1:** Global Quality Score

Score	Description
1	Poor quality, poor flow of the site, most information missing, not at all useful for patients
2	Generally poor quality and poor flow, some information listed but many important topics missing, of very limited use to patients
3	Moderate quality, suboptimal flow, some important information is adequately discussed but others poorly discussed, somewhat useful for patients
4	Good quality and generally good flow, most of the relevant information is listed, but some topics not covered, useful for patients.
5	Excellent quality and excellent flow, very useful for patients

**Table 2:** Reliability Score For each question, yes is scored as 1 and no as 0. The sum is calculated.

Description
Are the aims clear and achieved?
Are reliable sources of information used?
Is the information presented balanced and unbiased?
Are additional sources of information listed for patient reference?
Does it refer to areas of uncertainty?

## Results

A total of 420 posts were included in this study. The number of posts under each hashtag that were analyzed and included in the study is shown in Table 3. 54.52% of posts were by the health and wellness industry, whereas only 17.86% of posts were by doctors. The characteristics of the post, i.e., type of post (image or video), engagement with viewers (likes and comments), and the type of accounts that created and uploaded the post are detailed in Table 4. There were 98 (23.33%) posts that had sponsored content. Table 5 presents the type of information about "obesity" that is present in these posts.

**Table 3:** Number of relevant posts under each hashtag

Hashtag names	Post analyzed	Relevant posts included in the study
#Obesity	100	72 (17.14%)
#Stopobesity	100	55 (13.09%)
#Obesityfreeindia	100	81 (19.28%)
#Obesitysurgery	100	46 (10.95%)
#Obesityawareness	100	72 (17.14%)
#Obesitysurvivor	100	74 (17.61%)
#Fightingobesity	100	20 (4.76%)
Total	700	420

**Table 4:** Characteristics of the post analyzed (n=420)

	N	Percentage of Total
Type of post		
Image/post	355	84.52
Video	65	15.48
Total number of audiences reached by the posts		
Absolute no. of likes	280,696	
Absolute no. of comments	5335	
Type of account that created and uploaded the post		
Doctor	75	17.86
Health and wellness industry/website	229	54.52
News agency	05	1.19
Pharmaceutical company	01	0.24
Dietician	27	6.43
Survivor/person suffering from the disease	58	13.81
Other	25	5.95

**Table 5:** Type of content being circulated about “obesity”

	N	%
Description about disease/surgery by doctor	68	16.19
Etiology	80	19.05
Prevalence	35	8.33
Symptoms	79	18.81
Diagnosis	27	6.43
Screening	39	9.29
Prevention	104	24.76
Treatment	82	19.52
Mortality	16	3.81
Rehabilitation	66	15.71
Support groups	99	23.57
People's own experience	130	3.95
Experience with family member	210	50
Promotional post by pharmaceutical company/doctor	98	23.33

The data were divided into two groups: group A (n = 304) consisted of information posted by doctors and others in the healthcare industry involved in active patient care (doctors, nurses, hospitals); group B (n = 116) included the rest of the sources. The two groups were compared for the percentage of correct information being circulated, and the difference was statistically significant using the Z-test. Table 6 depicts the Global Quality Score and Reliability Score of the posts analyzed, and Table 7 shows that the percentage of correct information is higher in group A than in group B, and this difference is statistically significant. Further comparisons were made between the two groups regarding the quality and reliability of the content being circulated. Although the GQS was higher in posts by doctors and the healthcare industry, this difference was not statistically significant. However, the reliability score of the posts by doctors and the healthcare industry was "significantly greater" than that of the other group (Table 8).

**Table 6:** Global Quality Score and Reliability Score of the posts analyzed

		N	%
Good quality score			
1	Very low	132	31.43
2	Low	120	28.57
3	Medium	90	21.43
4	High	63	15.00
5	Very high	15	3.57
Reliability score			
1	Posts with score 1	162	38.57
2	Posts with score 2	136	32.38
3	Posts with score 3	72	17.14
4	Posts with score 4	36	8.57
5	Posts with score 5	14	3.33

**Table 7:** Comparison of correct information being circulated between two groups P-value: 0.000532 (p < 0.05; significant) Z score: −3.273050

	Group A (n= 304)	Group B (n= 116)
No. of correct posts	167	43
Percentage	54.93%	37.07%

**Table 8:** Comparison of quality and reliability of posts between two groups

	Group A (n= 304)	Group B (n= 116)
Global Quality Score		
Mean ± SD	2.35 ±1.21	2.21 ± 1.03
P-value = 0.2708		
Reliability Score		
Mean ± SD	2.12 ± 1.16	1.91 ± 0.87

## Discussion

YouTube has been a source for sharing information, not just about obesity but about several healthcare diseases like stroke [6-9], asthma [10,11], myocardial infarction [12], and breast cancer [13,14].

The 420 posts included in this study had a wide reach - 280,696 likes and 5335 comments - signifying that they reached a large population. The total number of people who engaged (reacted) with this post can be estimated by the number of likes and comments. However, the actual reaction (who viewed the post) can be much larger, as not everyone engages with the post. In a study by Clancy et al. [6] concerning the access to the internet and social media by low-morbidity stroke survivors participating in a national web-based secondary stroke prevention program, the authors found that almost 79% of participants used the Internet at least daily, 40% accessed social media on their phone or tablet daily, and 46% looked up health and medical information at least monthly.

In our study, 17% of the posts were uploaded by doctors and 54% by the health and wellness industry (Table 4). This is similar to a study by Szmuda et al. [7] on patient information about stroke on YouTube, wherein 65% of the videos were uploaded by hospitals, and over 60% of them had a doctor speaking. It is necessary that healthcare-related information be posted on social media platforms like YouTube only by doctors and hospitals that are certified to do so. This will significantly reduce misinformation.

The promotional content posted by pharmaceutical companies in our study was 24%. In a study by Heineman et al. [10] on asthma therapies, the researchers found that 42% of the information was promotional, and of that, only 32% was consistent with the Global Initiative for Asthma guidelines, leaving a significant amount of information lying around that was not supported by the Global Initiative for Asthma. This is a favorable finding because it is necessary that information about healthcare-related conditions is not driven by the promotional strategies of pharmaceutical companies, which might try to promote their own products using social media platforms, thereby modifying the decision-making capacities of the patients and preventing accurate information transmission to them.

In our study, 25% of posts were about the prevention of obesity, while a study by Pant et al. [12] on assessing the credibility of YouTube's approach to health information on acute myocardial infarction reported that 17% of the videos were giving away information about prevention by stressing weight loss and exercise programs, and these were ultimately advertisements for specific weight loss programs and diet pills. Both of these conditions are preventable, if not curable, and yet the percentage of videos communicating about prevention is lower. If verified sources such as doctors and hospitals upload more posts about these conditions, it will hugely impact patients’ viewers' towards adapting the correct prevention strategies and even following the prescribed treatment measures. In a study by Thackeray et al. [13] on using Twitter for breast cancer prevention, it was found that most of the tweets did not advocate for any specific preventive actions. Twitter was mostly used as a one-way messaging platform. Organizations require a strategic communications plan to ensure ongoing social media interactions in order to increase the message's reach and the potential for word-of-mouth marketing via Twitter. In these discussions, organizations might think about cooperating with commoners and famous people.

In our study, 20% of the posts were about treatment, 19% were about sharing information about etiology, and similarly, 19% were about explaining the symptoms. Posts that were in the form of experiences from family members were about 50%, while 23% of the posts were from support groups. The Global Quality Score and reliability score turned out to be high for only a small 9%, indicating the lesser reliability of the majority of posts. In a study by Askin et al. [8] on quality, reliability, and accuracy analysis for YouTube as a source of information for transcranial magnetic stimulation in stroke, most of the videos, i.e., 47%, were found to be of intermediate quality and had partial sufficient data.

However, on dissecting and comparing the number of correct posts among the types of people uploading, it was found that doctors and the health and wellness industry had a significantly higher number of correct posts, i.e., about 43%. A study by Diers et al. [11] on asthma showed that only 7.7% of the content was uploaded by lung specialists and 65% of the overall videos had correct information. In another study by Brachtenbach et al. [14] about searching for answers on breast cancer on YouTube, researchers found that 22.5% of the videos scored a mean point of 7 or more, 9% scored a mean of 0 or fewer, and another 9% of the videos in their sample contained at least one misleading statement, with nine of those having multiple misleading statements.

Limitations

The posts that were not in English or Hindi were excluded. Each person was only assigned 10 posts per day, owing to the fact that an average person scrolling Instagram would generally not look beyond 10 posts about obesity as it tends to get overwhelming. Also, the calculation of the Global Quality Score and Reliability Score may vary from person to person.

## Conclusions

The videos uploaded by doctors and the healthcare industry on YouTube related to diabetes are more accurate and reliable than those from other sources. In conclusion, our study highlights the need for continued monitoring and evaluation of the spread of healthcare information on social media platforms. This is important in ensuring that users have access to accurate and reliable information, which is crucial in their health decision-making. Future research in this area should focus on developing strategies to promote the dissemination of accurate healthcare information and counter the spread of misinformation on social media platforms. Additionally, further studies are needed to determine the long-term impact of misinformation on public health and inform public health policies that aim to mitigate this impact.
